# Genetic mechanisms of bone digestion and nutrient absorption in the bone-eating worm *Osedax japonicus* inferred from transcriptome and gene expression analyses

**DOI:** 10.1186/s12862-016-0844-4

**Published:** 2017-01-13

**Authors:** Norio Miyamoto, Masa-aki Yoshida, Hiroyuki Koga, Yoshihiro Fujiwara

**Affiliations:** 1 Japan Agency for Marine-Earth Science and Techonology, Yokosuka, Kanagawa Japan; 2 National Institute of Genetics, Mishima, Shizuoka Japan; 3 Postodoctral research fellow, Japanese Society for Promotion of Science, Tokyo, Japan; 4 Graduate School of Life and Environmental Sciences, University of Tsukuba, Tsukuba, Ibaraki Japan

**Keywords:** Matrix metalloproteinase, Bone digestion, Solute carrier family transporter, Nutrient uptake

## Abstract

**Background:**

Bone-eating worms of the genus *Osedax* (Annelida, Siboglinidae) have adapted to whale fall environments by acquiring a novel characteristic called the root, which branches and penetrates into sunken bones. The worms lack a digestive tract and mouth opening, and it has been suggested that *Osedax* degrade vertebrate bones and uptake nutrients through acidification and secretion of enzymes from the root. Symbiotic bacteria in the root tissue may have a crucial role in the metabolism of *Osedax*. However, the molecular mechanisms and cells responsible for bone digestion and nutrient uptake are still unclear, and information on the metabolic interaction between *Osedax* and symbiotic bacteria is limited.

**Results:**

We compared transcriptomes from three different RNA samples from the following tissues: trunk + palps, root + ovisac, and larva + male. A Pfam domain enrichment analysis revealed that protease- and transporter-related genes were enriched in the root + ovisac specific genes compared with the total transcriptome. Through targeted gene annotation we found gene family expansions resulting in a remarkably large number of matrix metalloproteinase (*mmp*) genes in the *Osedax* compared with other invertebrates. Twelve of these *Osedax mmp* genes were expressed in the root epidermal cells. Genes encoding various types of transporters, including amino acid, oligopeptide, bicarbonate, and sulfate/carboxylate transporters, were also expressed in root epidermal cells. In addition, amino acid and other metabolite transporter genes were expressed in bacteriocytes. These protease and transporter genes were first expressed in root tissues at the juvenile stage, when the root starts to develop.

**Conclusions:**

The expression of various proteinase and transporter genes in the root epidermis supports the theory that the root epidermal cells are responsible for bone digestion and subsequent nutrient uptake. Expression of transporter genes in the host bacteriocytes suggests the presence of metabolic interaction between *Osedax* and symbiotic bacteria.

**Electronic supplementary material:**

The online version of this article (doi:10.1186/s12862-016-0844-4) contains supplementary material, which is available to authorized users.

## Background

The deep sea is one of the few remaining frontiers in the field of biology. Since the discovery of an invertebrate community in the Galapagos Rift in 1977 [1], several chemosynthetic ecosystems have been found in hydrothermal vents and hydrocarbon seeps worldwide [2, 3]. In these environments, many endemic species, such as vestimentiferan tubeworms, vesicomyid clams, and *Rimicaris* shrimps, have been reported [2]. These organisms have evolved to consume new nutrient sources, with chemosynthetic energy obtained through symbiosis with chemosynthetic microbes (reviewed in [4, 5]). In addition to vents and seeps, another type of deep-sea community has also been discovered, referred to as the whale-fall ecosystem [6]. When a carcass of a large vertebrate (e.g., a whale) sinks to the sea floor, the huge source of organic material harbors a variety of organisms. Initially, mobile scavengers such as sharks, hagfishes, and crustaceans aggregate and consume the soft tissue of the carcass [7]. After the bones of the carcass are exposed, enigmatic marine worms of the genus *Osedax* colonize on the bones [8].

**Fig. 1:**
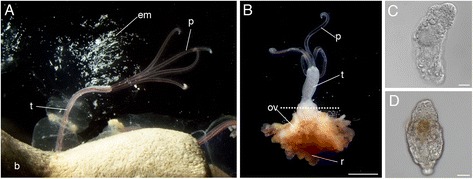
Photographs *Osedax japonicus*. **a** An adult female living on a bone. White spots around the female are embryos spawned into transparent mucus. **b** An adult female exposed on a bone. The dot line indicates the position that was cut for RNA extraction. Upper and lower fragments are trunk palps (trunk) and root + ovisac (root), respectively. **c** A dwarf male. **d** A larva with settlement competency. b, bone; em, embryo; ov, ovisac; p, palp; r, root; t, trunk. Scale bars: B = 2 mm; C and D = 20 μm

*Osedax* are marine invertebrates that belong to the phylum Annelida, family Siboglinidae [8]. They exclusively inhabit sunken whale bones under natural conditions and are able to colonize bones of other vertebrate species under experimental conditions [9–11]. Since their discovery, at least 27 higher taxonomic units have been described worldwide [8, 11–19]. *Osedax* usually shows remarkable sexual dimorphism, with vermiform females (Fig. 1a, b), and microscopic dwarf males (Fig. 1c) [8]. *Osedax* has trochophore-type larvae (Fig. 1d). Adult females of *Osedax* consist of four regions: palps, trunk, ovisac, and root. By consuming vertebrate bones, *Osedax* worms play a role in the degradation of sunken body remains and the recycling of deep-sea carbon [15, 20]. The female *Osedax* lacks a digestive tract, including a mouth, gut, and anus. Instead, it has been suggested that they use their posterior root system as their digestive organ. The posteriorly branching root system is an evolutionary novel organ [8, 20, 21], which penetrates into bones and contains heterotrophic bacteria enclosed in bacteriocytes (Fig. 1a, b). It has been suggested that the microvillar root cells secrete digestive enzymes and acid to aid in the degradation and uptake of nutrients from the dissolved bones [22–25]. Although it has been assumed that these endosymbiotic bacteria have a function in the metabolism of nutrients, the specific process remains unclear [20, 23, 26, 27]. To address the evolution of *Osedax* worms, an understanding of the origin and detailed function of the root is necessary. A recent study has shown that the root epidermal cells are immunoreactive against anti-vascular proton ATPase (V-H^+^ ATPase) and anti-carbonic anhydrase (CA) antibodies [24]. This suggests that V-H^+^ ATPase are responsible for dissolving the calcium phosphate in vertebrate bones through acidification of the microenvironment surrounding the root [24]. Another study, which showed that the root of *Osedax* has collagenolytic activity, suggested that the root tissues that include symbiotic bacteria secrete enzymes to digest the matrix proteins of bones [23]. The absence of matrix proteinase gene in the symbiont genomes suggests that host worms are responsible for collagen degradation [27]. However, it is not known what varieties of enzymes are secreted from what type of cells, and the mechanisms behind nutrient uptake.

Here, we performed transcriptome analysis and examined the spatial and developmental expression patterns of genes related to digestion and nutrient uptake in the bone-eating worm *Osedax japonicus*, which can be reared for multiple generations under laboratory conditions [28]. Comparing the transcriptomes from the three different tissues: root + ovisac (root), trunk + palp (trunk), and larva + male, we identified the genes that were specifically expressed in the root. We investigated the expression patterns of genes of interest that were considered related to bone digestion and nutrient uptake. The results revealed novel aspects of the molecular mechanisms of the metabolic process of *Osedax* worms.

## Results

### Sequencing of *O. japonicus* transcriptome

We sequenced three types of RNA samples: trunk, root, and larva + male (Fig. 1a–d) using a HiSeq2000 sequencer (Illumina, San Diego, CA, USA). Males of *O. japonicus* are dwarf males and are approximately 500 μm in length (Fig. 1c). Owing to the small amounts of total RNA obtained from males and larvae, these two sample types were combined for sequencing. A total of 200,523,188 reads were obtained and the basic information regarding the raw data and assembly is shown in the supporting information (Additional file 1: Figure S1, Additional file 2: Table S1). The raw reads were assembled into 57,194 contigs and 38,913 subcomponents by Trinity software [29] with a contig N50 of 1383 bp. Although the read assembly was still incomplete, we considered these subcomponents to be distinct genetic loci. We set the threshold for gene expression intensity (fragments per kilobase per million reads, [FPKM] ≥1, see Methods) to eliminate fluctuations or background noise and 22,541 genes were found above the threshold in at least one sample. Among 22,541 genes, the numbers of trunk, root, and larva + male-specific genes were 825, 1214, and 5485, respectively. The 22,541 genes were compared against UniprotKB with BLASTX and 9448 genes were homologous to known functional genes. Among these 9448 genes, 185, 223, and 1258 genes were trunk, root, and larva + male specific, respectively. The list of root specific gene is shown in the supporting information (Additional file 3: Figure S2, Additional file 4: Table S2). Raw data are available in the DDBJ sequence Read Archive (DRA003880).

### Characterization of the root transcriptome

To elucidate the features of *O. japonicus* transcriptome, we attempted functional annotation of novel sequence data using gene ontology (GO) annotation and a single enrichment analysis against the total *O. japonicas* transcriptome. GO term enrichment analysis showed that 11, nine, and six terms associated with “molecular function,” “cellular component,” and “biological process,” respectively, were significantly over-represented in the root-specific genes against all gene set backgrounds (Additional file 5: Table S3). The over-represented GO terms included hydrolase activity, transferase activity, transport, and catalytic activity, which were all related to the nutrient uptake and metabolic processes. Only two terms of “molecular function” and a term of “biological process” were under-represented in the root transcriptome (Additional file 5: Table S3).

We also looked for significantly enriched Pfam domains [30] in the proteins encoded by the root-specific genes against the total transcriptome (Table 1). In the root-specific genes, we found 36 enriched Pfam domains (Table 1). Matrixin (PF00413), hemopexin (PF00045), putative peptidoglycan binding domain (PF01471), and peptidase families (PF05649, PF01433, and PF01431) are components of matrix metalloproteinase (MMP) proteins, which degrade the extracellular matrix (ECM) including various types of collagen. Pfam domains related to other types of proteases were also enriched in the root-specific gene set. The peptidase family M13 (PF05649) is a domain found in membrane metalloendopeptidase (MME), which cleaves peptides at the amino side of hydrophobic residues. Zinc carboxypeptidase (PF00246) and carboxypeptidase activation peptide (PF02244) are domains related to carboxypeptidase activity that hydrolyze peptides at the carboxyl-terminal end of proteins and peptides. We also found an enrichment of domains related to substrate transportation. The amino acid permeases (PF13520 and PF00324) domain is a component of amino acid transporters belonging to the solute carrier (SLC) family transporters, such as the SLC7 and SLC12 families. We found that three domains related to another member of the SLC transporter family were enriched in the root transcriptome. Sodium:sulfate symporter transmembrane region (PF00939) and citrate transporter (PF03600) are components of the SLC13 family Na^+^-sulfate/carboxylate cotransporter. The domain related to a sugar transporter (PF00083) was also enriched in the root-specific gene set. The ABC transporter transmembrane region (PF00664) and ABC transporter (PF00005) are domains related to the ATP-binding cassette (ABC) transporter, which translocates a variety of substrates.

**Table 1:** Pfam domains enriched in the root transcriptome

Pfam ID	Domain description	Total	Root	*P*.value	Q.value
PF00413.19	Matrixin	62	26	5.96E-22	8.10E-20
PF00045.14	Hemopexin	40	20	4.45E-19	3.02E-17
PF01471.13	Putative peptidoglycan binding domain	31	14	5.95E-13	2.69E-11
PF05649.8	Peptidase family M13	34	12	8.85E-10	3.01E-08
PF13520.1	Amino acid permease	21	7	4.94E-06	1.12E-04
PF07690.11	Major Facilitator Superfamily	123	16	6.63E-06	1.29E-04
PF00939.14	Sodium:sulfate symporter transmembrane region	10	5	1.17E-05	1.76E-04
PF03600.11	Citrate transporter	10	5	1.17E-05	1.76E-04
PF00324.16	Amino acid permease	24	7	1.34E-05	1.82E-04
PF00246.19	Zinc carboxypeptidase	27	7	3.14E-05	3.88E-04
PF07953.7	Clostridium neurotoxin, N-terminal receptor binding	7	4	4.93E-05	5.15E-04
PF12388.3	Dual-action HEIGH metallo-peptidase	7	4	4.93E-05	5.15E-04
PF14670.1	Coagulation Factor Xa inhibitory site	68	10	1.27E-05	1.23E-03
PF01433.15	Peptidase family M1	24	6	1.47E-04	1.33E-03
PF14653.1	Insulin growth factor-like family	16	5	1.70E-04	1.44E-03
PF01431.16	Peptidase family M13	36	7	2.24E-04	1.79E-03
PF02244.11	Carboxypeptidase activation peptide	6	3	8.07E-04	5.77E-03
PF00431.15	CUB domain	46	7	1.06E-03	7.20E-03
PF05572.8	Pregnancy-associated plasma protein-A	7	3	1.37E-03	8.90E-03
PF01607.19	Chitin binding Peritrophin-A domain	63	8	1.60E-03	9.88E-03
PF13582.1	Metallo-peptidase family M12B Reprolysin-like	27	5	2.27E-03	1.29E-02
PF00664.18	ABC transporter transmembrane region	41	6	2.95E-03	1.53E-02
PF10462.4	Peptidase M66	9	3	3.13E-03	1.53E-02
PF00209.13	Sodium:neurotransmitter symporter family	32	5	4.91E-03	2.22E-02
PF13385.1	Concanavalin A-like lectin/glucanases superfamily	80	8	7.12E-03	2.93E-02
PF00005.22	ABC transporter	66	7	8.43E-03	3.37E-02
PF13688.1	Metallo-peptidase family M12	24	4	9.29E-03	3.58E-02
PF01391.13	Collagen triple helix repeat (20 copies)	39	5	1.15E-02	3.58E-02
PF03209.10	PUCC protein	5	2	1.16E-02	3.58E-02
PF04727.8	ELMO/CED-12 family	5	2	1.16E-02	3.58E-02
PF11669.3	WW domain-binding protein 1	5	2	1.16E-02	3.58E-02
PF13243.1	Prenyltransferase-like	5	2	1.16E-02	3.58E-02
PF11838.3	ERAP1-like C-terminal domain	14	3	1.19E-02	3.58E-02
PF00629.18	MAM domain	26	4	1.24E-02	3.58E-02
PF00012.15	Hsp70 protein	15	3	1.45E-02	4.10E-02
PF00083.19	Sugar (and other) transporter	43	5	1.71E-02	4.65E-02

### Expression of protease genes in the root

To identify cells responsible for protease secretion, we examined expression pattern of genes encoding protease, which degraded the ECM. Because Pfam domains related to the MMP protein were enriched in the root-specific gene set, we first examined the expression of *mmp* genes. From the transcriptome of all samples, 24 *mmp* genes containing the catalytic domain with a conserved zinc-binding motif were found. Among them, 22 *mmp* genes were detected from the root transcriptome and 13 *mmp* genes were expressed only in the root (Additional file 6, Table S4). We examined the expression of 16 *mmp* genes and performed double staining with a probe against the *16S rRNA* gene of *Neptunomonas japonica* (*Nj 16S rRNA*) infected with *O. japonicus* to clarify the localization of bacteriocytes harboring symbiotic bacteria.

**Fig. 2:**
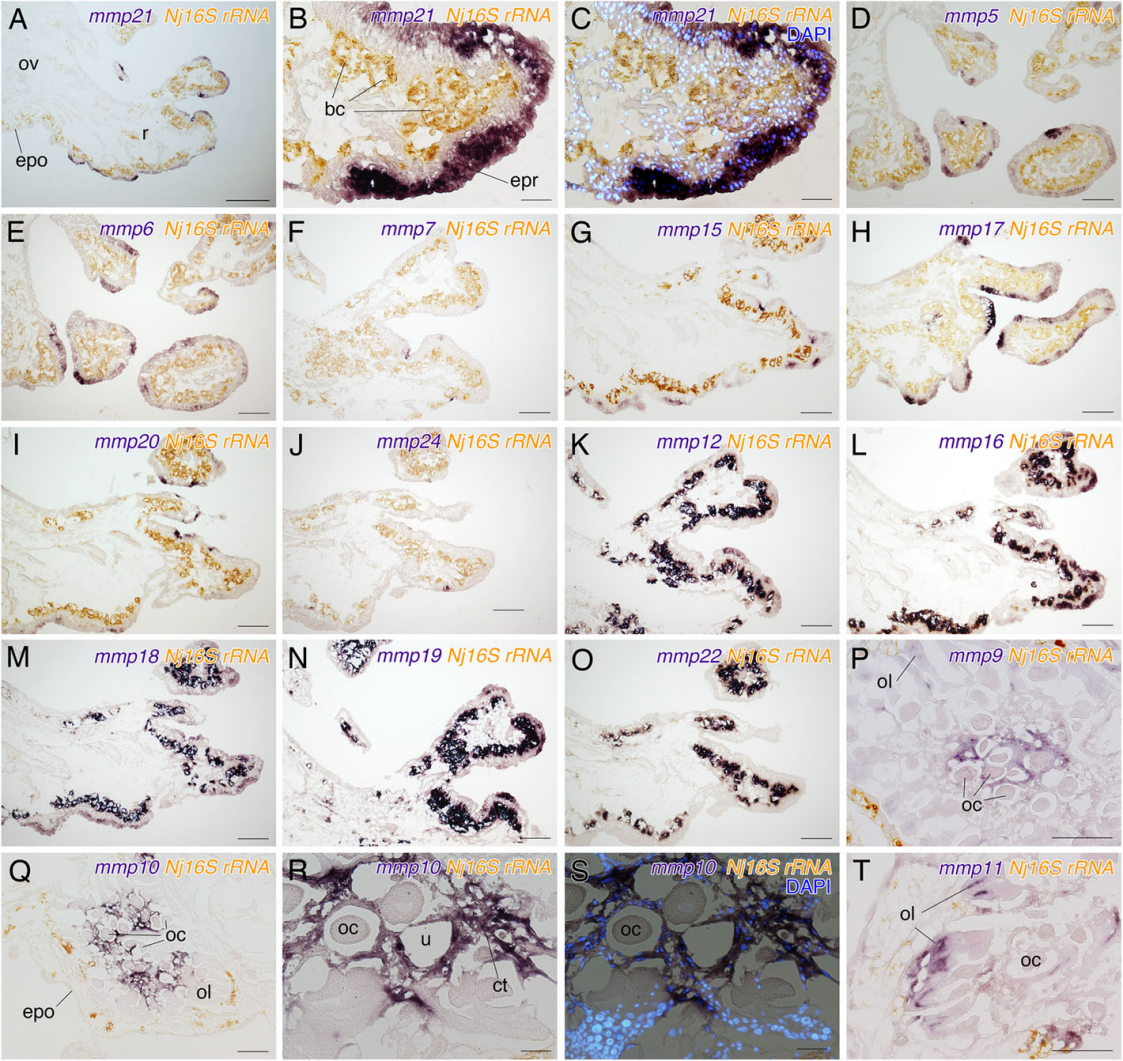
Expression patterns of *mmp* genes in *Osedax japonicus*. Purple signals are expression of *mmp* genes and yellow signals are expression of *16S rRNA* of *Neptunomonas japonica*. Sagittal sections. Anterior is top. **a**–**c** Broad epidermal expression of *Oja-mmp21*. **d**–**j** Expression patterns of *Oja-mmp5*, *Oja-mmp6*, *Oja-mmp7*, *Oja-mmp15*, *Oja-mmp17, Oja-mmp20*, and *Oja-mmp24* in the epidermis of the root. **k**–**n** Expression patterns of *Oja-mmp12*, *Oja-mmp16*, *Oja-mmp18*, and *Oja-mmp19* in the root epidermis and bacteriocytes. **o** Expression of *Oja-mmp22* in bacteriocytes. **p** Expression of *Oja-mmp9* in follicle cells and connective tissue surrounding oocytes. **q**–**s** Expression of *Oja-mmp10* was detected in the connective tissue surrounding oocytes. Counterstaining with DAPI indicated that *Oja-mmp10* was also expressed in follicle cells. **t** *Oja-mmp11* expression detected in the germinal epithelium between the stalks of developing oocytes. bc, bacteriocyte; ct, connective tissue; epo, ovisac epidermis; epr, root epidermis; oc, oocyte; ol, ovarian lobe; ov, ovisac; r, root; u, uterus. Scale bars: A = 500 μm; B, C, R, S = 50 μm; D–Q, T = 200 μm

Eight of the 16 *mmp* genes were solely expressed in the epidermis of the root (Fig. 2a–j): *Oja-mmp5*, *Oja-mmp6*, *Oja-mmp7*, *Oja-mmp15*, *Oja-mmp17*, *Oja-mmp20*, *Oja-mmp21*, and *Oja-mmp24*. The details of the expression patterns in these genes differed. *Oja-mmp21* was most broadly expressed (Fig. 2a–c). Counterstaining with 4′,6-diamino-2-2phenylindole (DAPI) showed that *Oja-mmp21* was exclusively expressed in epidermal cells (Fig. 2c). With the exception of *Oja-mmp17*, all of these genes were specific to the root transcriptome (Additional file 7: Table S4) and positive signals were detected only in the peripheral branching region of the root and not in the epidermis of the ovisac. Doublestaining with *Nj 16S rRNA* indicated that five *mmp* genes, *Oja-mmp12*, *Oja-mmp16*, *Oja-mmp18*, *Oja-mmp19*, and *Oja-mmp22* were expressed in bacteriocytes (Fig. 2k–o) with all but *Oja-mmp22* also expressed in the root epidermis (Fig. 2k–n). Three genes, *Oja-mmp9*, *Oja-mmp10*, and *Oja-mmp11* were solely expressed in the internal tissues related to the reproductive system. The expression of *Oja-mmp9* and *Oja-mmp10* were detected in the epithelial cells of ovarian tissue (Fig. 2p, q). Expression of *Oja-mmp9* was detected only in the epithelial cells of the uterus [31] surrounding mature oocytes. Counterstaining with DAPI showed that *Oja-mmp10* was also expressed in connective tissues around the uterus (Fig. 2r, s). In *O. japonicus*, the ovarian lobe consists of two rows of previtellogenic oocytes. Between the two rows of previtellogenic oocytes there are thin cells and *Oja-mmp11* expression was observed in these cells (Fig. 2t).

**Fig. 3:**
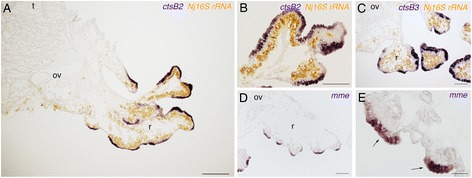
Expression of protease genes. **a**, **b** Expression of *Oja-ctsB2* detected in root epidermis. **c** *Oja-ctsB3* expressed in root epidermis. **d**, **e** *Oja-mme* expressed in root epidermal cells. Positive signals were detected only in thick cells. ov, ovisac; r, root; t, trunk. Scale bars: A = 500 μm; B–D = 200 μm; E = 50 μm

Other varieties of proteases were also found in the root transcriptome. Three cathepsin Bs (*ctsB* genes: *Oja-ctsB1*, *Oja-ctsB2*, and *Oja-ctsB3*) were identified in *Osedax. Oja-ctsB2* and *Oja-ctsB3* were present exclusively in the root transcriptome, whereas *Oja-ctsB1* was detected in all three transcriptomes (Additional file 6: Table S4). The expressions of *Oja-ctsB2* and *Oja-ctsB3* were detected in the root epidermis (Fig. 3a–c). The expression patterns of *Oja-ctsB2* and *Oja-ctsB3* were almost identical, with both genes expressed in the epidermis of the distal part of the branching root. No signal was detected at the proximal part, which is close to the ovaries. The expressions of *Oja-ctsB2* and *Oja-ctsB3* were not ubiquitous in root epidermis. There were negative cells in the distal part of the root, which were thin, whereas the positive regions were thick (Fig. 3a–c). MME is a type II transmembrane glycoprotein and a neutral endopeptidase that belongs to the peptidase family M13. As mentioned above, the peptidase family M13 domain was enriched in the root transcriptome (Table 1). An *mme* gene, *Oja-mme* was present only in the root transcriptome (Additional file 6: Table S4) and expressed in root epidermal cells (Fig. 3d, e).

### Expression of transporter genes in the root

SLC family transporters play crucial roles in the uptake and efflux of compounds such as sugars, amino acids, nucleotides, inorganic ions, and fatty acids [32, 33]. In the *O. japonicus* transcriptome, 40 families in the *slc* gene superfamily, with at least 233 genes, were identified (Additional file 8: Table S5). Although the *slc* gene nomenclature system has been defined in mammals [33], due to low resolution of the phylogenetic analyses and the likely lineage-specific molecular evolution of the *slc* genes, the genes were named in an *Osedax*-specific manner.

**Fig. 4:**
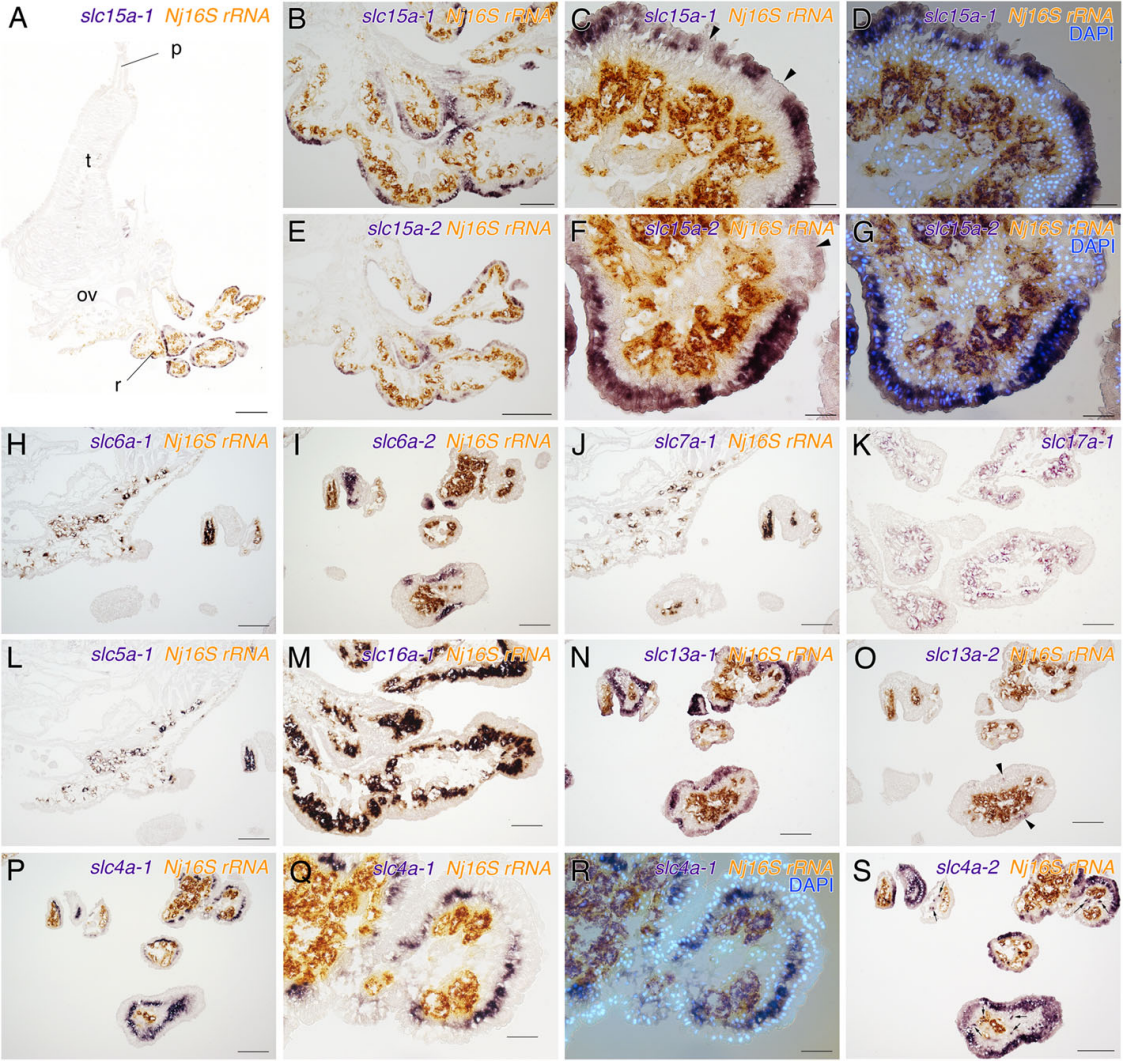
Expression patterns of *slc* genes in *Osedax japonicus*. **a**–**d** Expression of an oligopeptide transporter gene *Oja-slc15a-1.* **a**, **b** Positive signals detected at the distal part of root tissue, where there is no ovisac beneath the epidermis. **c**, **d** Counterstaining with DAPI showed that *Oja-slc15a-1* was expressed only in root epidermal cells. **e**–**g** Expression of another oligopeptide transporter gene *Oja-cls15a-2.* **f**, **g** A high-magnification image and counterstaining with DAPI indicated that *Oja-slc15a-2* was expressed in root epidermal cells. **h** Double-staining with *Nj16S rRNA* showed an amino acid transporter gene *Oja-slc6a-1* expressed in bacteriocytes. **i** An amino acid transporter gene *Oja-slc6a-2* was expressed in root epidermal cells. **j** Double-staining with *Nj 16S rRNA* showed that an amino acid transporter gene *Oja-slc7a-1* was expressed in bacteriocytes. **k** Expression of an amino acid transporter gene *Oja-slc17a-1* was detected in bacteriocytes. **l** Double-staining with *Nj 16S rRNA* indicated that a glucose transporter gene *Oja-slc5a-1* was expressed in bacteriocytes. **m** Double-staining with *Nj 16S rRNA* indicated that a monocarboxylate transporter gene *Oja-slc16a-1* was expressed in bacteriocytes. **n**, **o** Expression of two carboxylate transporter genes, *Oja-slc13a-1* and *Oja-slc13a-2*. Expression of *Oja-slc13a-1* was detected broadly in root epidermis, whereas expression of *Oja-slc13a-2* was detected only in restricted cells of the root epidermis (arrowheads). **p**–**s** Expression patterns of two bicarbonate transporter genes, *Oja-slc4a-1* and *Oja-slc4a-2*. **q**, **r** Counterstaining with DAPI indicated that *Oja-slc4a-1* was expressed in mesodermal cells underlying the epidermis of the root. **s** *Oja-slc4a-2* expression detected in root epidermal cells. The gene was also expressed in mesenchymal cells in the root (arrow). ov, ovisac; p, palp; r, root; t, trunk. Scale bars: A = 500 μm; B, E, H–Q, S = 200 μm; C, D, F, G, R = 50 μm

The SLC15 family members are proton-coupled oligopeptide transporters in vertebrates [34]. In the *O. japonicus* root transcriptome, two genes of the SLC15 family, *Oja-slc15a-1* and *Oja-slc15a-2*, were found. *Oja-slc15a-2* was detected only from the root transcriptome. These two genes were expressed in the epidermis of the root (Fig. 4a–g). In the root, expression was restricted to the peripheral branching regions and no signal was detected in the epidermis of the ovisac (Fig. 4a). In the distal part of the root, there were some cells in which a positive signal was not detected (Fig. 4c, f, arrowheads). A nuclear counter stain with DAPI showed that these genes were expressed exclusively in the epidermis (Fig. 4d, g). The members of the SLC6 family are Na^+^- and Cl^-^-dependent neurotransmitter symporters that translocate small amino acid or amino acid-like substrates [35]. Expression of *Oja-slc6a-1* was detected in bacteriocytes where it overlapped a positive *Nj 16S rRNA* signal (Fig. 4h). *Oja-slc6a-2* was also expressed in the root epidermis (Fig. 4i). Expression was not widespread in the epidermis, being restricted to small populations of epidermal cells (Fig. 4i). Transporters of the SLC7 family are amino acid transporters [36]. In *O. japonicus*, a *slc7* gene, *Oja-slc7a-1*, was expressed in bacteriocytes (Fig. 4j). The SLC17 family has been described as vesicular glutamate transporters [37]. In *O. japonicus*, a positive *Oja-slc17a-1* signal overlapped with that of *Nj 16S rRNA*. This expression indicated that *Oja-slc17a-1* was expressed in bacteriocytes (Fig. 4k).

The expression patterns of other metabolite transporters were also examined. The members of the SLC5 family are glucose transporters that play an important role in sugar uptake [38]. In *O. japonicus*, *Oja-slc5a-1* was expressed in the bacteriocytes (Fig. 4l). The SLC16 family members are monocarboxylate transporters that catalyze the proton-linked transport of monocarboxylates such as lactate, pyruvate and ketone bodies [39]. A member of the *slc16* family gene, *Oja-slc16a-1*, was expressed in the bacteriocytes (Fig. 4m). The SLC13 family members are Na^+^ and SO_4_^2-^-carboxylate co-transporters, which translocate anions, or di- and tri-carboxylate citric acid cycle intermediates in vertebrates [40]. Two *slc13* genes, *Oja-slc13a-1* and *Oja-slc13a-2*, were expressed in the root epidermis (Fig. 4n, o). Expression of *Oja-slc13a-1* was widespread in root epidermis (Fig. 4n), whereas *Oja-slc13a-2* was expressed only in a small number of root epidermal cells (Fig. 4o, arrowheads). The SLC4 family of bicarbonate transporters transfers the bicarbonate ion or a related species across the plasma membrane [41]. *Oja-slc4a-1* was expressed in the mesodermal cells in the root (Fig. 4p). A nucleus counterstain with DAPI detected positive *Oja-slc4a-1* signals in the cell layer just underneath the epidermis, which was identified as a muscle layer (Fig. 4q, r, [26]). *Oja-slc4a-2* was expressed in the root epidermis and non-symbiotic mesenchymal cells around bacteriocytes (Fig. 4s, arrows).

### Expression of protease and transporter genes in the larval and juvenile stages

**Fig. 5:**
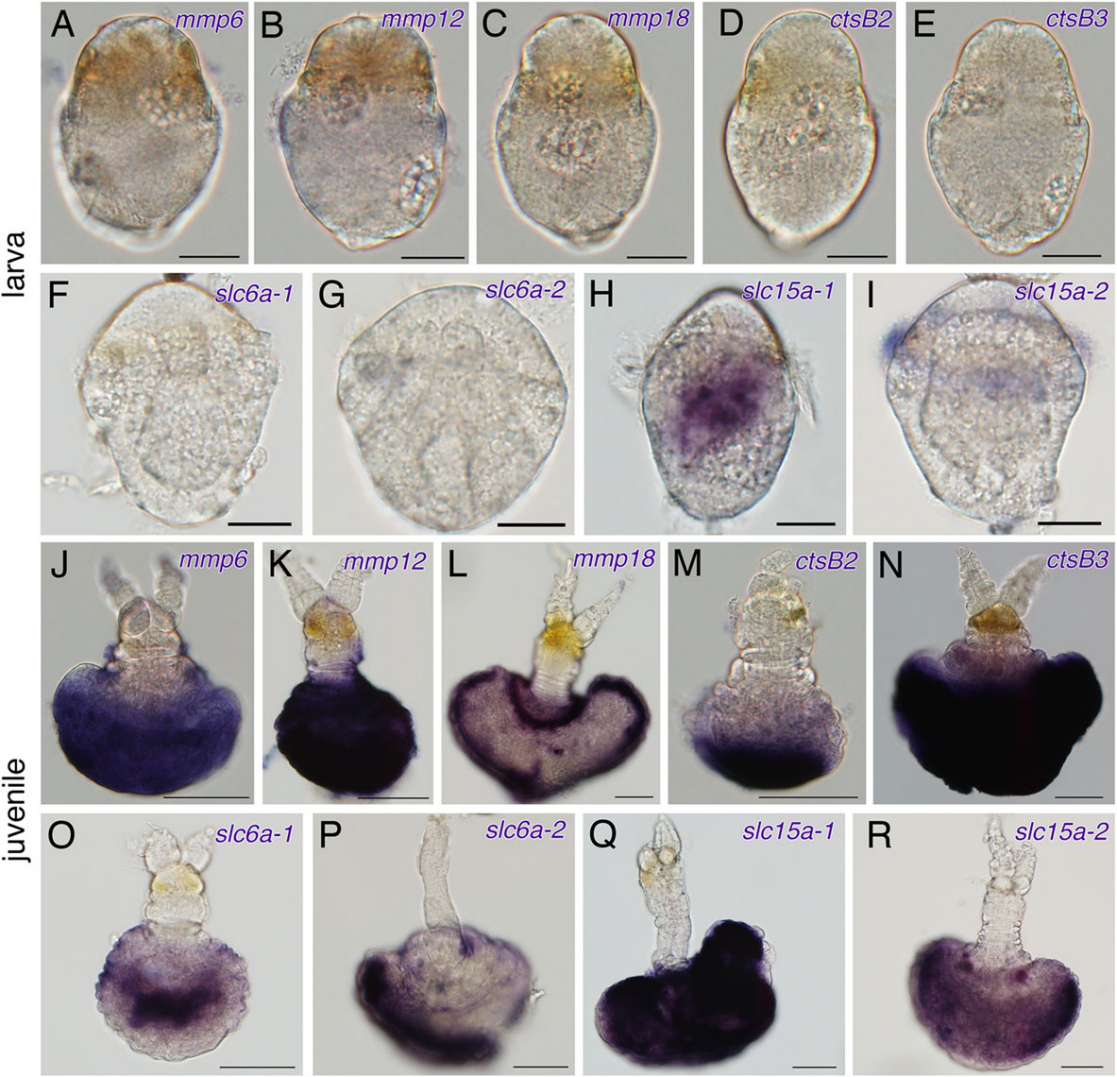
Expression patterns of protease and transporter genes in larvae and juveniles of *Osedax japonicus. Oja-mmp6*, *Oja-mmp12*, *Oja-mmp18*, *Oja-ctsB2*, *Oja-ctsB3*, *Oja-slc6a-1*, *Oja-slc6a-2, Oja-slc15a-1* and *Oja-slc15a-2* expression in larvae (**a**–**i**) and juveniles (**j**–**r**). At the larval stage, only expression of *Oja-slc15a-1* was detected in the endoderm. **j**–**l** Three *mmp* genes, *Oja-mmp6*, *Oja-mmp12*, and *Oja-mmp18* were expressed in the root epidermal cells. **m**, **n** Expression of *Oja-ctsB2* was detected in the posterior part of the root epidermis, whereas expression of *Oja-ctsB3* was detected widely in root epidermal cells. **o** Expression of *Oja-slc6a-1* was detected inside cells of the root, where bacteriocytes were located. **p** *Oja-slc6a-2* expression was detected in part of the root epidermis. **q**, **r** Expression of *Oja-slc15a-1* and *Oja-slc15a-2* were detected in root epidermal cells. Scale bars = 50 μm

For the larval stages, expressions of *Oja-mmp6*, *Oja-mmp12*, *Oja-mmp18*, *Oja-ctsB2*, *Oja-ctsB3*, *Oja-slc6a-1*, *Oja-slc6a-2*, and *Oja-slc15a-2* genes were not detected (Fig. 5a–g, i). *Oja-slc15a-1* was expressed in the internal tissue of larvae (Fig. 5h). However all of these genes were expressed in the root tissue at the juvenile stage, 2–5 days after settlement (Fig. 5j–r). *Oja-mmp6*, *Oja-mmp12*, *Oja-mmp18*, *Oja-ctsB3*, *Oja-slc15a-1*, and *Oja-slc15a-2* were expressed widely in the root epidermis of juveniles (Fig. 5j, k, l n, q, r). *Oja-ctsB2* was expressed in the posterior part of the developing root, whereas *Oja-ctsB3* was expressed widely in the root (Fig. 5m, n). A positive *Oja-slc6a-1* signal was detected inside the root where bacteriocytes were present (Fig. 5o). *Oja-slc6a-2* was expressed in part of the root epidermis (Fig. 5p).

## Discussion

### Bone digestion by proteases

Vertebrate bones are made of both organic and inorganic components. One of the main inorganic components is calcium phosphate. A previous study reported that *Osedax* root epidermis cells are immunoreactive against anti-V-H^+^ ATPase and anti-CA antibodies [24]. The V-H^+^ ATPase and CA are also expressed in the osteoclasts in vertebrates and are involved in the resorption of bones through acidification [42]. Vertebrate bones and cartilage also contain many different organic components, with the majority being ECM proteins, such as various types of collagens. MMPs are a large family of zinc dependent endoproteinases and are able to degrade ECM components [43]. Vertebrates have seven types of fibrillar collagens, encoded by eleven genes [44]. Types I, V, and XXIV are components of mineralized bone, whereas types II, XI, and XXVII are components of cartilage [44]. In this study, we found at least 24 MMPs in *O. japonicus*. Of these genes, 12 were expressed in the root epidermis. The number of *mmp* genes is small in invertebrates (two in the fly *Drosophila melanogaster* and seven in the ascidian *Ciona intestinalis*, [45]), except for in the sea urchin ([46]; 26 in the purple sea urchin *Strongylocentrotus purpuratus*). In vertebrates, at least 25 different *mmp* genes have been identified with 24 of them present in humans [47]. In spiralians, little is known about *mmp* genes. We surveyed the genomes of three spiralians: the pacific oyster *Crassostrea gigas* [48], the owl limpet *Lottia gigantea*, and a marine polychaete *Capitella teleta* [49], and found six, eight and five *mmp* genes, respectively.

This remarkable increase of MMPs followed by amino acid substitutions and expression in the root epidermis in *O. japonicus* most likely enables them to digest various types of ECMs in vertebrate bones. In addition to ECM degradation in bone and cartilage, MMPs play essential roles in morphogenesis, immune system functioning, and reproduction through the remodeling of ECMs in vertebrates and invertebrates [50, 51]. We detected expression of some *Osedax mmp* genes in root epidermis, bacteriocytes, and ovarian tissues. The peculiar shape of the root tissues implies that their morphogenesis is regulated by the repeated growth and remodeling of ECMs. Some MMPs may also have a function during the formation of the root tissue.

**Fig. 6:**
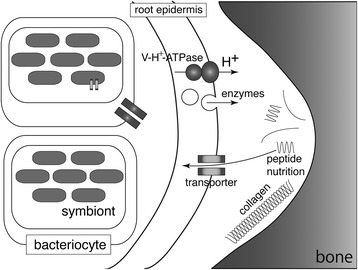
Schematic illustration of bone digestion and nutrient uptake in *Osedax japonicus*. Calcium phosphate in vertebrate bone is digested through acidification of the microenvironment by V-H^+^-ATPase [24]. Organic components of bones, such as collagens are degraded by proteases secreted from root epidermis. The digested peptides and other nutrients contained in the bones are transported through various membrane transporters. Some of the nutrients are transported to symbiotic bacteria, which use the nutrients as a metabolic resource

In vertebrates, cathepsin K (CTSK) is highly expressed in the osteoclast and is responsible for the digestion of type I collagen, which is the main component of mineralized bone [52, 53]. In *O. japonicus*, we could not find CTSK in the transcriptome, but three *ctsB* genes were expressed in the root. CTSB is a papain-like cysteine protease that is usually located in the lysozome in normal cells and tissues in humans [54]. In the clam *Meretrix meretrix* and the shrimp *Pandalus borealis*, CTSB are expressed in the digestive tract (digestive gland and hepatopancreas, respectively) and have been reported to have a possible role in nutrient digestion [55, 56]. We showed that some protease genes, *mmps* and *ctsBs*, were expressed at the early juvenile stage when the worms start to bore into bones, whereas no expression was detected during the larval stage. The expression patterns of protease genes in the host worm, together with the absence of collagenase genes in *Osedax* symbionts [27], strengthen the possibility that the root epidermal cells play critical roles in the digestion of bone matrix proteins (Fig. 6).

### Nutrient uptake and metabolic interaction between host and symbiotic bacteria

The SLC superfamily is the largest group of membrane transporter proteins, and includes 52 families and 395 transporter genes in the human genome [33]. The SLC transporters play essential roles in nutrient uptake in the intestine as well as in the translocation of various kinds of molecules in other tissues and cell types. The functions of each member of the SLC transporter are well characterized in humans [33]; however, little is known about the function of SLC transporters in invertebrates. Almost all families of the SLC transporters are conserved among bilaterians [57]. In this study, we confirmed the expression of some *slc* genes encoding oligopeptide and amino acid transporters in the root epidermis of *O. japonicus*. In mammals, members of the proton-coupled oligopeptide transporter family (SLC15) are responsible for the absorption of dietary protein digestion products in the intestine [34]. In *O. japonicas*, we found that two genes encoding SLC15 family transporters were expressed in the root epidermis. Root epidermal cells started to express one of two *slc15* genes at the early juvenile stage, when the animals began a sessile lifestyle and started consuming vertebrate bones. We also detected expression of a gene encoding the amino acid transporter family (SLC6) in the root epidermis. These transporters are the candidate molecules playing a crucial role in nutrient uptake. Further functional analysis will unveil the molecular mechanisms for digestion and absorption by the root of *Osedax* worms.

In this study, we found the expression of genes encoding amino acid, glucose, and monocarboxylate transporters in bacteriocytes. The genome sequence of *Osedax* symbionts showed that they encode a number of transporter genes for various substrates such as amino acids, peptides, and carbohydrates [27]. The exact function of symbiotic bacteria in *Osedax* worms is still unclear; however, expression and possession of transporter genes in the host bacteriocytes and symbiont genome suggest the presence of metabolic interaction between *Osedax* and symbiotic bacteria (Fig. 6).

## Conclusions

Because there is no comparable counterpart close relative of *Osedax*, the root is considered an evolutionary novelty. Here, we investigated tissue-specific transcriptome and the expression pattern of genes, which appear to be related to the function of the root. Expression of various proteinase including *mmp* and transporter genes in the root epidermis support the theory that the root epidermal cells are responsible for bone digestion and subsequent nutrient uptake. Expression of amino acid, sugar and other metabolite transporter genes in the host bacteriocytes suggests the presence of metabolic interaction between *Osedax* and symbiotic bacteria. Further studies using the *O. japonicus* and *N. japonica* system, which allows us to examine the biology of both species and the interactions between them, should provide essential information regarding the evolutionary novelty of *Osedax.*

## Methods

### Animal collection and laboratory culture

We obtained *O. japonicus* specimens from whale bones collected at a depth of 226 m off Cape Noma Misaki via a remotely operated vehicle (ROV), *Hyper-Dolphin*, on the research vessel (R/V) *Natsushima* on March 28 and April 13, 2012. *Osedax japonicus* specimens were kept in 100-L tanks at 11 °C in the laboratory together with whale bones. Embryos and males were collected from the mucus of females. Larval settlement was induced by adding small pieces of vertebrate bone to dishes where the larvae were kept [28]. To induce the infection of *N. japonica*, we incubated the bones with *N. japonicus* overnight at 20 °C. After a brief wash with filtered artificial sea water, the bones were cultured with larvae.

### RNA extraction and sequencing

We extracted total RNA from three samples: the female trunk + palps (trunk), the root + ovisac (root), and larvae + adult males, using QIAzol reagent followed by column purification using RNeasy Mini Kit (Qiagen, Limburg, Netherlands) according to the manufacturer’s instructions. The quality and concentration of total RNA was analyzed by gel electrophoresis and a NanoDrop Spectrophotometer (Agilent, Santa Clara, CA, USA), respectively. Only samples that met the manufacturer’s criteria (BGI: http://www.bgi.com/us/services/genomics/whole-transcriptome-sequencing/) were then processed for the RNA-seq analysis. Short read sequences were obtained using a Hiseq2000 sequencer (Illumina, San Diego, CA, USA) according to the manufacturer’s procedures. High quality bases (quality score ≥20) were used in following analyses. The raw sequence data are available in the DDBJ sequence Read Archive (DRA003880).

### Bioinformatic analyses

Three fastq files obtained from the samples were combined into a dataset and *de novo* assembly of the *O. japonicus* transcriptome without a reference genome was performed using Trinity (version r2013-02-25) [29]. To obtain normalized gene expression intensities (in FPKM), reads from each of the three samples were mapped onto the Trinity assembly with the bowtie [58], and analyzed with the RNA-seq by Expectation-Maximization (RSEM: version 1.2.3; [59]) and edgeR [60]. In the assembly procedure, putative alternative splicing variants were estimated as different contigs per sub-component; however we merged variants from one sub-component based on the “%comp_fpkm” values of the RSEM output when we estimated the tissue specificity of each sub-component. The Maser analytical pipelines on the National Institute of Genetics Cell Innovation program (http://cell-innovation.nig.ac.jp/) were used for the following functional estimations of the assembled Trinity contigs.

GO enrichment analysis was employed to identify GO terms associated with a subset of genes and to test whether this association (enrichment) is significantly different from what would be expected by chance given a background gene set (in this case, the entire gene set). GO terms were assigned to nucleotide contigs of the *Osedax* assembly against a set of the protein database of UniprotKB that showed BLASTX hits with a threshold E-value of 10^-10^. FatiGo was employed for the statistical enrichment test of root specific genes against the pool of all transcriptome based on the annotated subcomponents after a false discovery rate < 0.01 correction [61].

For the protein annotation analyses of *Osedax* sequences, the longest open reading frames and corresponding amino acid sequences per subcomponents were predicted using a Perl script, with sequences longer than 30 amino acids used for downstream analysis. Transmembrane domains and signal sequence regions were predicted with TMHMM 2.0 [62] and SignalP 4.1 [63]. Pfam domains were assigned based on the results of a HMMER (3.1b2; [64]) search against the Pfam-A database [30], with a threshold E-value of 10^-5^. The statistical analyses of the Pfam enrichment analysis were performed using R (3.1.2), with the Q-value package (1.43.0; [65]). The *p*-values of the enriched domains in the root-specific gene set against the pool of three transcriptomes were calculated using a hypergeometric distribution, with a false discovery rate < 0.05 correction. The Pfam enrichment analyses were performed using domains that were assigned to at least five subcomponents.

### Phylogenetic analyses of genes

For the analysis counterpart of *Osedax*, we searched for *mmp* genes from three spiralians genomes already published: the pacific oyster *Crassostrea gigas* [48], the owl limpet *Lottia gigantea*, and a marine polychaete *Capitella teleta* [49], using BLAST on the genome browsers of the animals. We first performed TBLASTN against each genome using *Osedax* MMP sequences as queries (an E-value of < 1e^−10^). Gene orthologies were confirmed by the phylogenetic analyses described later.

Fragments of genes were amplified by PCR (the primer sequences and accession numbers are in Additional file 9: Table S6). Gene orthologies were inferred by maximum likelihood analyses and multiple alignments. Amino-acid alignments were made with MAFFT ver. 7 [66]. Sequences were trimmed by trimAl [67]. Maximum likelihood analyses were performed with RAxML ver. 8 [68].

### In situ hybridization

Animals were fixed with 4% paraformaldehyde (PFA) in Mops buffer (0.1 M Mops, 0.5 M NaCl) at 4 °C, overnight. After several washes with PBST (i.e., PBS containing 0.1% Tween 20), samples were dehydrated through an ethanol series and stored in 80% ethanol at –20 °C. We used two in situ hybridization methods for visualizing spatiotemporal gene expression patterns. For the whole-mount in situ hybridization of larvae and juvenile females, samples were rehydrated and washed three times with PBST. The samples were digested with 1 μg/ml proteinase K/PBST for 20 min at 37 °C. After a brief wash with PBST, the samples were postfixed in 4% PFA/PBST for 10 min at room temperature (20–25 °C, RT), and washed three times with PBST. The samples were prehybridized in hybridization solution (50% formamide, 5× SSC, 5× Denhardt’s solution, 100 μg/ml yeast RNA, and 0.1% Tween 20) at 55 °C for 2 h and hybridized with a hybridization solution containing a digoxigenin (DIG)-labeled RNA probe at 55 °C for at least 16 h. For the negative control, an RNA probe of a gene, which was not expressed in the stages examined, was used (data not shown). The samples were washed with a solution of 50% formamide, 4× SSC, and 0.1% Tween 20 for 30 min twice; 50% formamide, 2× SSC, and 0.1% Tween 20 for 30 min twice; 2× SSC and 0.1% Tween 20 for 30 min twice; and 0.2× SSC and 0.1% Tween 20 for 30 min twice at 55 °C. The samples were washed with MABT (i.e., maleic acid buffer containing 0.1% Tween 20) three times, blocked in 2% blocking reagent (Roche, Indianapolis, IN, USA) in MABT for 60 min at RT, and incubated overnight at 4 °C with a 1:1500 dilution of anti-DIG-AP antibody (Roche) in the blocking buffer. Samples were then washed six times with MABT for 60 min and transferred into AP buffer (100 mM Tris pH 9.5, 100 mM NaCl, 50 mM MgCl_2_, and 10% dimethylformamide) without MgCl_2_ and dimethylformamide. After being washed twice with AP buffer, a chromogenic reaction was performed using nitro blue tetrazolium chloride/5-bromo-4-chloro-3-indolyl-phosphate (NBT/BCIP; Roche) in AP buffer until signals were visible. The reaction was stopped in PBST, postfixed in 4% PFA/PBST, rewashed with PBST, and mounted with 40% glycerol before being observed under a light microscope (IX71, Olympus).

For frozen sections, bones containing adult *O. japonicas* females were fixed overnight with 4% PFA/MOPS at 4 °C. The fixed samples were washed three times with PBS. We digested bones overnight with Morse’s solution at RT [69]. After we had picked worms from the bones, they were washed three times with PBS and mounted in Tissue-Tek® O.C.T compound to use as frozen sections. The in situ hybridization protocol of the frozen sections of adult females was based on a previous report [70]. For double staining, we used a fluorescein-labeled probe for hybridization. After the chromogenic reaction with NBT/BCIP described above, slides were incubated in 0.1 M glycine-HCl for 30 min to inactivate the alkaline phosphatase. Slides were washed three times with MAB, blocked in 2% blocking reagent in MAB for 60 min at RT, and incubated for 1 h at RT with a 1:2000 dilution of anti-fluorescein-AP antibody (Roche) in blocking buffer. They were washed six times with MAB for 60 min and transferred into AP buffer without MgCl_2_. After being washed twice with AP buffer, a chromogenic reaction was performed with 2-(4-iodophynyl)-5-(4-nitrophenyl)-3-pheniltetrazolium chloride/5-bromo-4-chloro-3-indolyl-phosphate (INT/BCIP; Roche) in AP buffer until signals were visible. The reaction was stopped in PBST, postfixed in 4% PFA/PBS, rewashed with PBS, and mounted with 80% glycerol. The slides were then observed under a light microscope.

## Additional files

Additional file 1: Figure S1. Gene expression intensity (fragments per kilobase per million reads) distribution of the *Osedax japonicus* transcriptome. (PDF 242 kb)
Additional file 2: Table S1. Summary of the transcriptome analysis. (XLSX 41 kb)
Additional file 3: Figure S2. Scatter plots of *Osedax japonicus* transcriptome. Root specific genes are indicated as magenta plots. Gray plots in the root specific area (X-axis < 0, Y-axis ≥ 0) are genes whose fragments per kilobase per million reads (FPKM) values of the other sample were above the threshold (≥0). For example, gray plots in the root specific area of the left graph are genes whose trunk FPKM values were above the threshold. (PDF 451 kb)
Additional file 4: Table S2. List of the root specific transcriptome. (XLSX 61 kb)
Additional file 5: Table S3. Significantly enriched gene ontology (GO) terms in the root transcriptome. (XLSX 45 kb)
Additional file 6: Table S4. Summary of *mmp* gene expression. (XLSX 47 kb)
Additional file 7: Figure S3. Phylogenetic trees of genes. The numbers at the nodes are bootstrap values (only those ≥ 50% are shown). Ac, *Aplysia californica*; Bb, *Branchiostoma belcheri*; Bf, *Branchiostoma floridae*; Cg, *Crassostrea gigas*; Ci, *Ciona intestinalis*; Ct, *Capitella teleta*; Dm, *Drosophila melanogaster*; Hs, *Homo sapiens*; Lg, *Lottia gigantea*; Mm, *Mus musculus*; Nv, *Nematostella vectensis*; Oj, *Osedax japonicus*; Pf, *Pinctada fucata*; Sk, *Saccoglossus kowalevskii*; Sp, *Strongylocentrotus purpurtus*; Tc, *Tribolium castaneum*. (PDF 528 kb)
Additional file 8: Table S5. Solute carrier (SLC) transporters in the *Osedax japonicus* transcriptome. (XLSX 31 kb)
Additional file 9: Table S6. Primer sets and accession numbers of genes. (XLSX 53 kb)
