# Supplementary figures and images for: Genetic mechanisms of bone digestion and nutrient absorption in the bone-eating worm Osedax japonicus inferred from transcriptome and gene expression analyses
Source: BMC Evol Biol. 2017 Jan 13;17:17. doi: 10.1186/s12862-016-0844-4 (PMC5237233; doi:10.1186/s12862-016-0844-4)

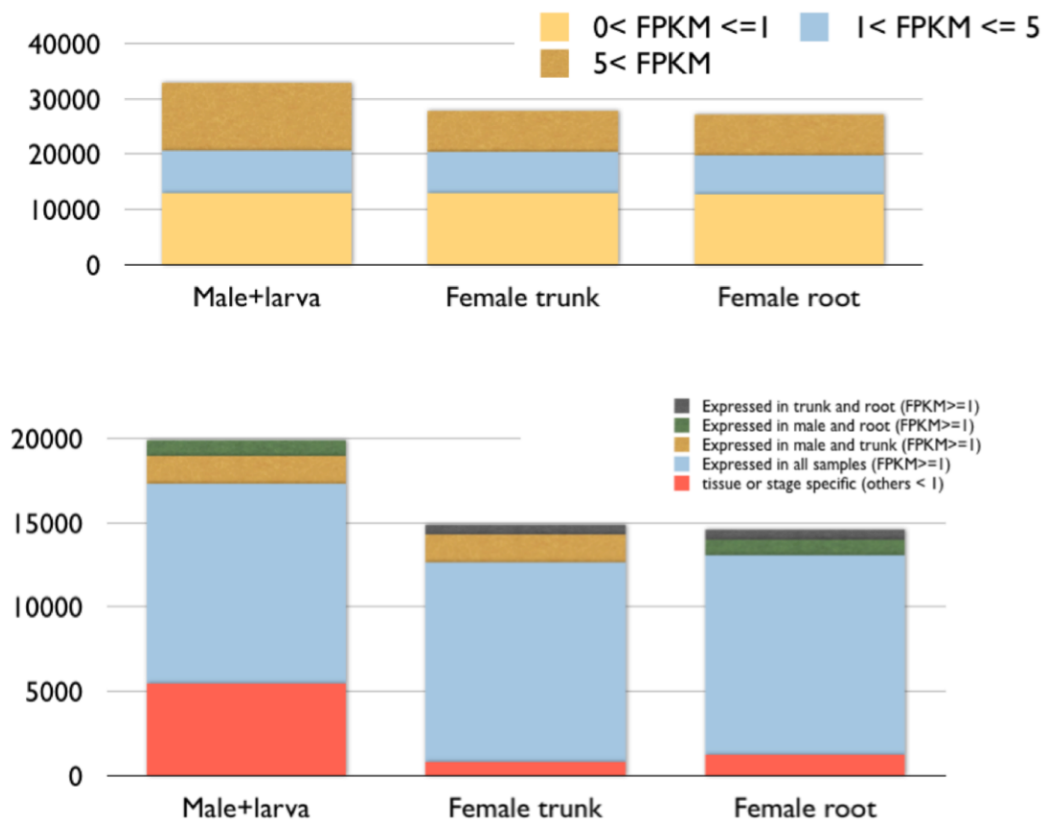

**Figure S1. FPKM distribution.**

Supplement: Additional file 1: Figure S1. — Gene expression intensity (fragments per kilobase per million reads) distribution of the Osedax japonicus transcriptome. (PDF 242 kb) [file 12862_2016_844_MOESM1_ESM.pdf]
