# Supplementary material for: Genetic mechanisms of bone digestion and nutrient absorption in the bone-eating worm Osedax japonicus inferred from transcriptome and gene expression analyses
Source: BMC Evol Biol. 2017 Jan 13;17:17. doi: 10.1186/s12862-016-0844-4 (PMC5237233; doi:10.1186/s12862-016-0844-4)
Supplement: Additional file 3: Figure S2. — Scatter plots of Osedax japonicus transcriptome. Root specific genes are indicated as magenta plots. Gray plots in the root specific area (X-axis < 0, Y-axis ≥ 0) are genes whose fragments per kilobase per million reads (FPKM) values of the other sample were above the threshold (≥0). For example, gray plots in the root specific area of the left graph are genes whose trunk FPKM values were above the threshold. (PDF 451 kb) [file 12862_2016_844_MOESM3_ESM.pdf]

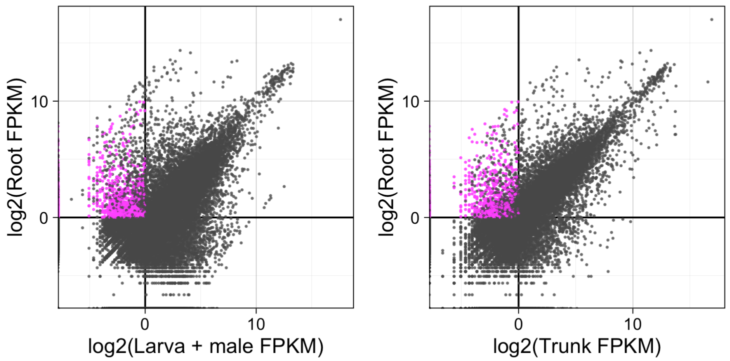

Figure S2. Scatter plots of *Osedax japonicus* transcriptome. Root specific genes are indicated as magenta plots. Gray plots in the root specific area (X-axis < 0, Y-axis ≥ 0) are genes whose fragments per kilobase per million reads (FPKM) values of the other sample were above the threshold (≥ 0). For example, gray plots in the root specific area of the left graph are genes whose trunk FPKM values were above the threshold.
