# Supplementary material for: Genetic mechanisms of bone digestion and nutrient absorption in the bone-eating worm Osedax japonicus inferred from transcriptome and gene expression analyses
Source: BMC Evol Biol. 2017 Jan 13;17:17. doi: 10.1186/s12862-016-0844-4 (PMC5237233; doi:10.1186/s12862-016-0844-4)
Supplement: Additional file 7: Figure S3. — Phylogenetic trees of genes. The numbers at the nodes are bootstrap values (only those ≥ 50% are shown). Ac, Aplysia californica; Bb, Branchiostoma belcheri; Bf, Branchiostoma floridae; Cg, Crassostrea gigas; Ci, Ciona intestinalis; Ct, Capitella teleta; Dm, Drosophila melanogaster; Hs, Homo sapiens; Lg, Lottia gigantea; Mm, Mus musculus; Nv, Nematostella vectensis; Oj, Osedax japonicus; Pf, Pinctada fucata; Sk, Saccoglossus kowalevskii; Sp, Strongylocentrotus purpurtus; Tc, Tribolium castaneum. (PDF 528 kb) [file 12862_2016_844_MOESM7_ESM.pdf]

a

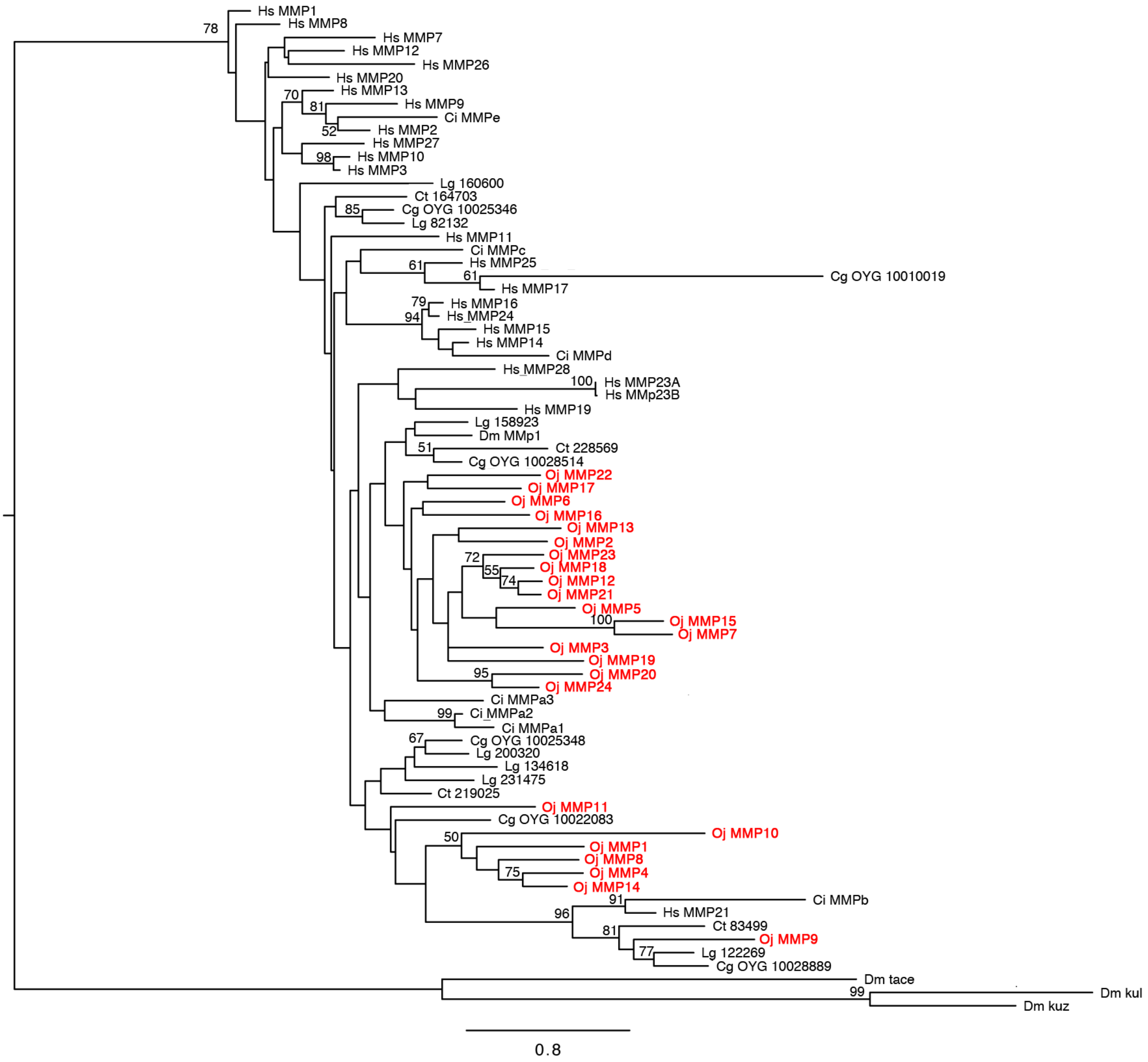
Hs MMP1
Hs MMP8
78
Hs MMP7
Hs MMP12
Hs MMP26
Hs MMP20
Hs MMP13
70
Hs MMP9
81
Ci MMPe
Hs MMP2
52
Hs MMP27
Hs MMP10
98
Hs MMP3
Lg 160600
Ct 164703
85
Cg OYG 10025346
Lg 82132
Hs MMP11
Ci MMPc
Hs MMP25
61
61
Cg OYG 10010019
Hs MMP17
79
Hs MMP16
94
Hs MMP24
Hs MMP15
Hs MMP14
Ci MMPd
Hs MMP28
100
Hs MMP23A
Hs MMp23B
Hs MMP19
Lg 158923
Dm MMp1
51
Ct 228569
Cg OYG 10028514
Oj MMP22
Oj MMP17
Oj MMP6
Oj MMP16
Oj MMP13
Oj MMP2
72
Oj MMP23
55
Oj MMP18
74
Oj MMP12
Oj MMP21
Oj MMP5
100
Oj MMP15
Oj MMP7
Oj MMP3
Oj MMP19
95
Oj MMP20
Oj MMP24
Ci MMPa3
99
Ci MMPa2
Ci MMPa1
67
Cg OYG 10025348
Lg 200320
Lg 134618
Lg 231475
Ct 219025
Oj MMP11
Cg OYG 10022083
50
Oj MMP10
Oj MMP1
Oj MMP8
75
Oj MMP4
Oj MMP14
91
Ci MMPb
Hs MMP21
96
81
Ct 83499
Oj MMP9
77
Lg 122269
Cg OYG 10028889
Dm tace
99
Dm kul
Dm kuz
0.8

b

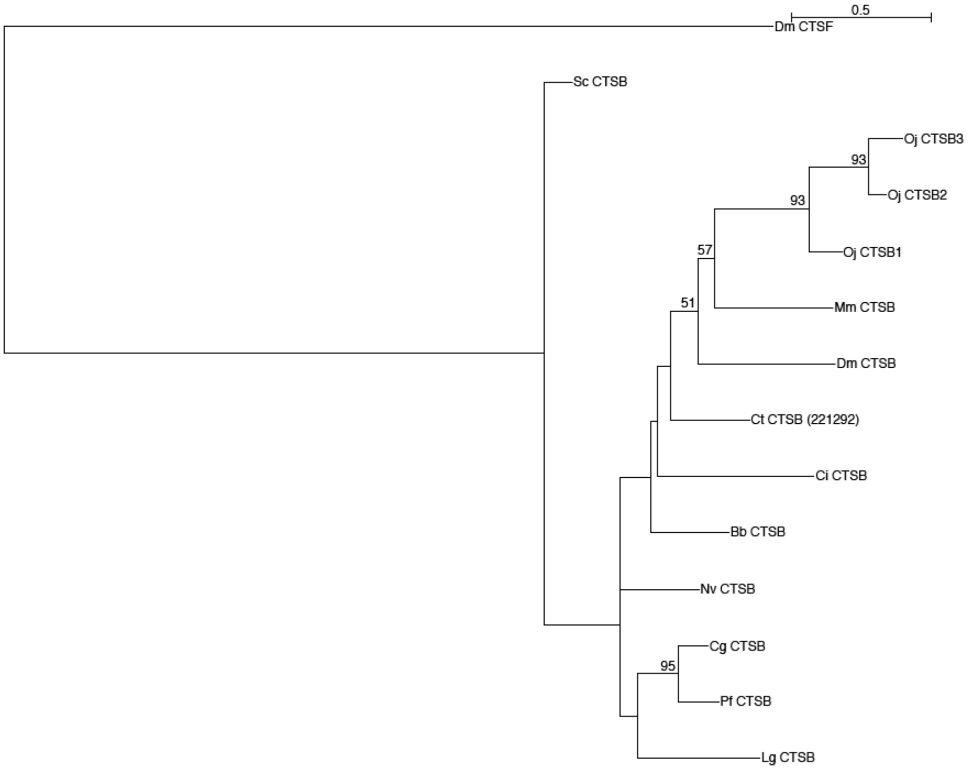
0.5
Dm CTSF
Sc CTSB
Oj CTSB3
93
Oj CTSB2
93
Oj CTSB1
57
Mm CTSB
51
Dm CTSB
Ct CTSB (221292)
Ci CTSB
Bb CTSB
Nv CTSB
Cg CTSB
95
Pf CTSB
Lg CTSB

c

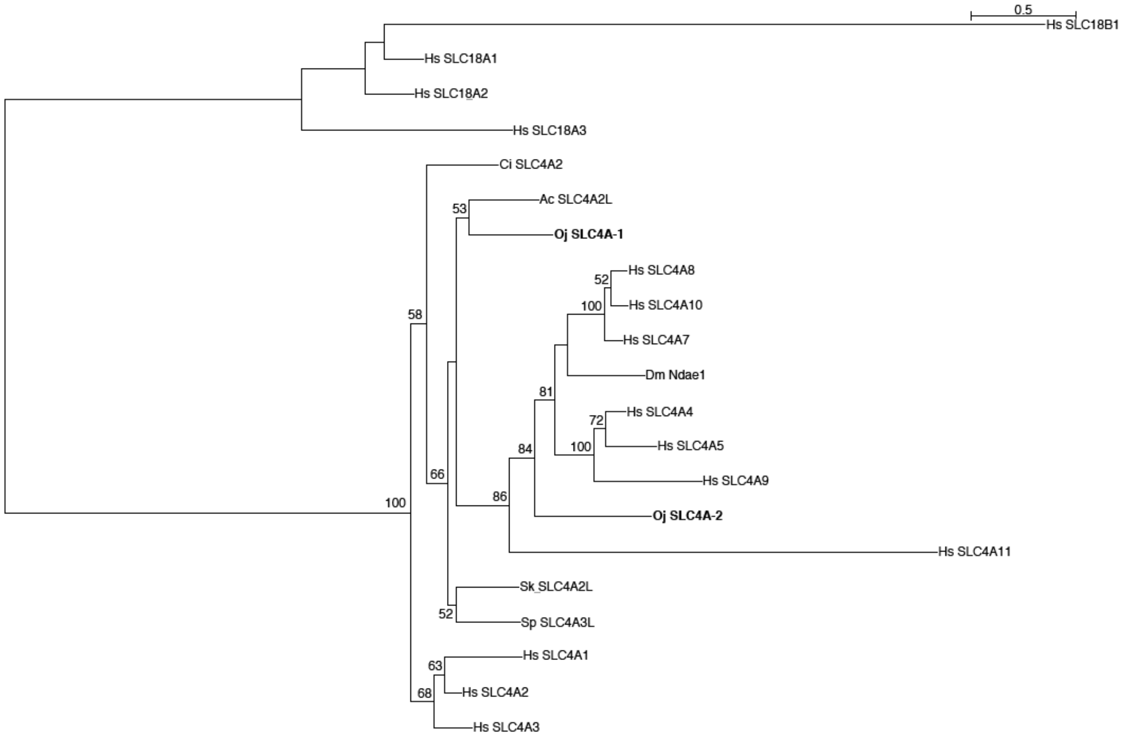
0.5
Hs SLC18B1
Hs SLC18A1
Hs SLC18A2
Hs SLC18A3
Ci SLC4A2
Ac SLC4A2L
53
Oj SLC4A-1
Hs SLC4A8
52
Hs SLC4A10
100
Hs SLC4A7
58
Dm Ndae1
81
Hs SLC4A4
72
Hs SLC4A5
100
84
Hs SLC4A9
66
86
Oj SLC4A-2
100
Hs SLC4A11
Sk SLC4A2L
52
Sp SLC4A3L
Hs SLC4A1
63
Hs SLC4A2
68
Hs SLC4A3

d

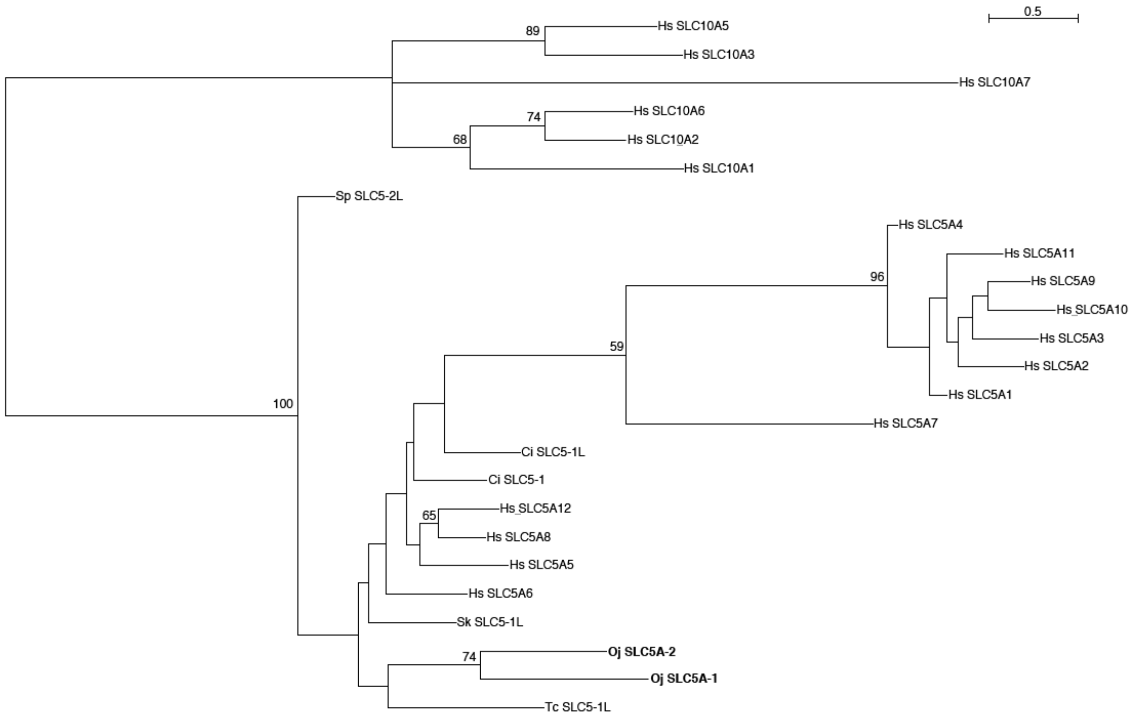
0.5
89
Hs SLC10A5
Hs SLC10A3
Hs SLC10A7
74
Hs SLC10A6
68
Hs SLC10A2
Hs SLC10A1
Sp SLC5-2L
Hs SLC5A4
Hs SLC5A11
96
Hs SLC5A9
Hs SLC5A10
Hs SLC5A3
59
Hs SLC5A2
Hs SLC5A1
100
Hs SLC5A7
Ci SLC5-1L
Ci SLC5-1
65
Hs SLC5A12
Hs SLC5A8
Hs SLC5A5
Hs SLC5A6
Sk SLC5-1L
74
Oj SLC5A-2
Oj SLC5A-1
Tc SLC5-1L

e

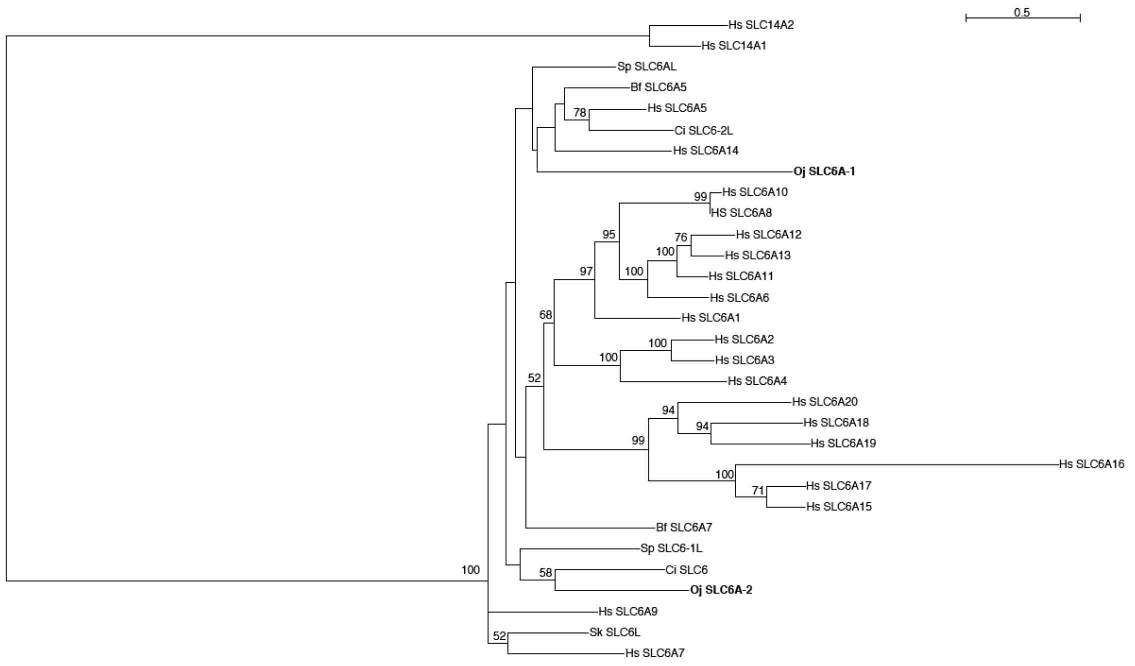
0.5
Hs SLC14A2
Hs SLC14A1
Sp SLC6AL
Bf SLC6A5
78
Hs SLC6A5
Ci SLC6-2L
Hs SLC6A14
Oj SLC6A-1
99
Hs SLC6A10
HS SLC6A8
95
76
Hs SLC6A12
100
Hs SLC6A13
97
100
Hs SLC6A11
Hs SLC6A6
68
Hs SLC6A1
100
Hs SLC6A2
100
Hs SLC6A3
Hs SLC6A4
52
Hs SLC6A20
94
94
Hs SLC6A18
99
Hs SLC6A19
100
Hs SLC6A16
71
Hs SLC6A17
Hs SLC6A15
Bf SLC6A7
Sp SLC6-1L
100
58
Ci SLC6
Oj SLC6A-2
Hs SLC6A9
52
Sk SLC6L
Hs SLC6A7

f

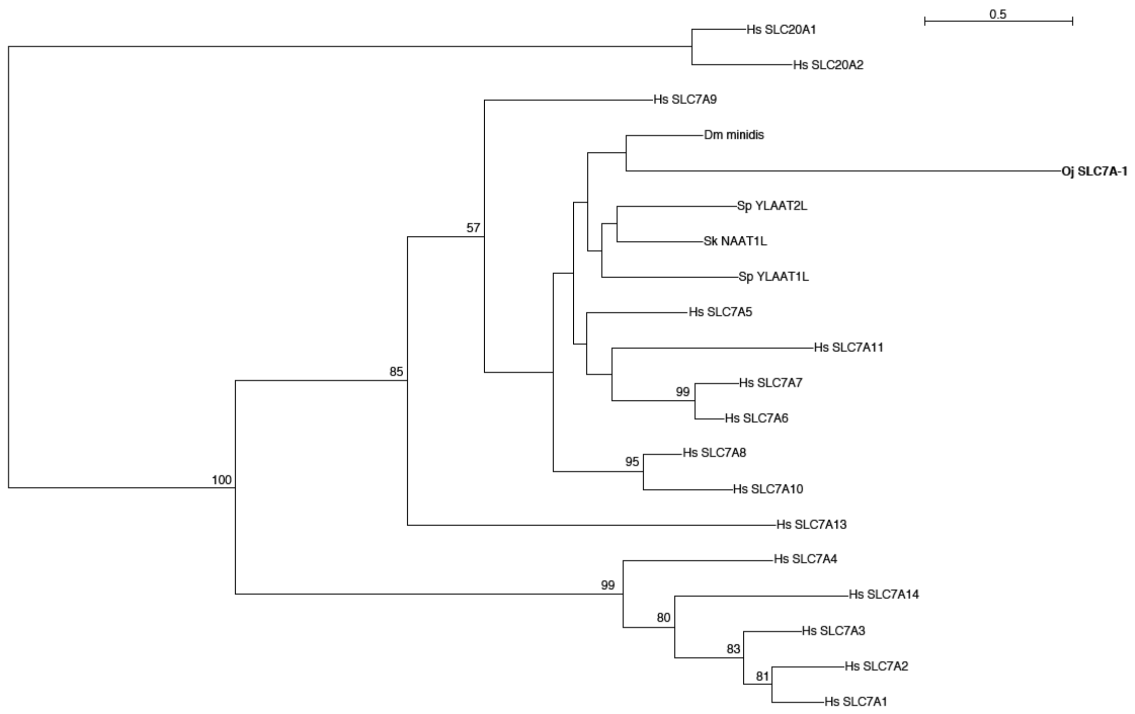
0.5
Hs SLC20A1
Hs SLC20A2
Hs SLC7A9
Dm minidis
Oj SLC7A-1
Sp YLAAT2L
Sk NAAT1L
Sp YLAAT1L
Hs SLC7A5
Hs SLC7A11
Hs SLC7A7
Hs SLC7A6
Hs SLC7A8
Hs SLC7A10
Hs SLC7A13
Hs SLC7A4
Hs SLC7A14
Hs SLC7A3
Hs SLC7A2
Hs SLC7A1
57
85
100
99
95
99
80
83
81

g

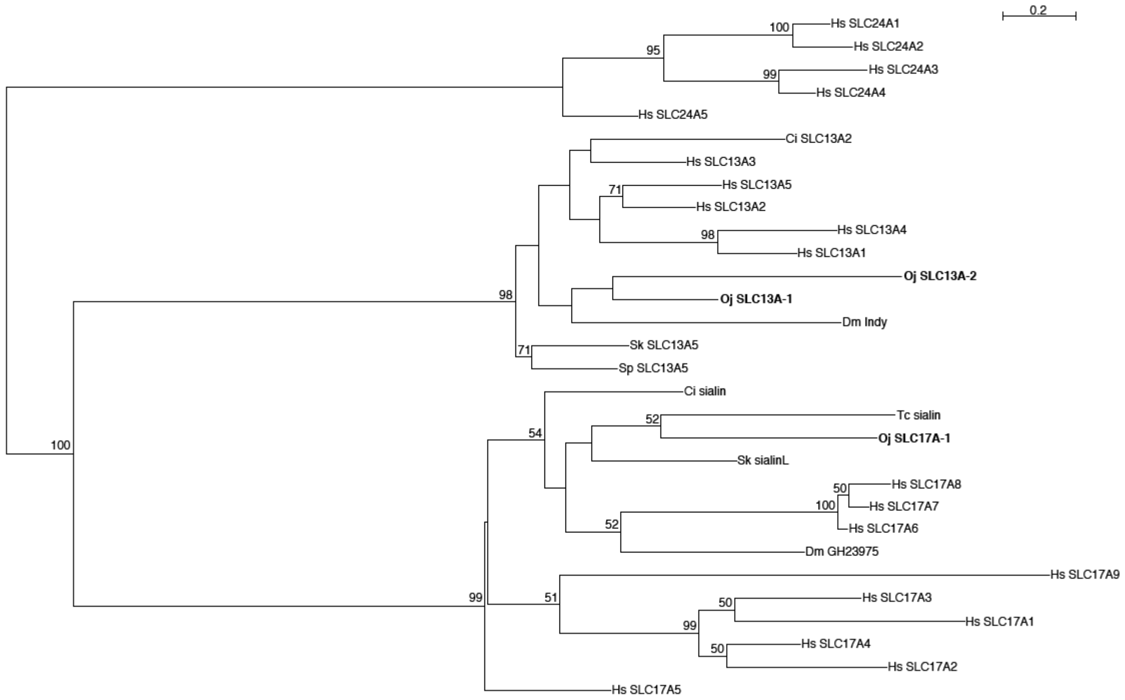
0.2
Hs SLC24A1
Hs SLC24A2
Hs SLC24A3
Hs SLC24A4
Hs SLC24A5
Ci SLC13A2
Hs SLC13A3
Hs SLC13A5
Hs SLC13A2
Hs SLC13A4
Hs SLC13A1
Oj SLC13A-2
Oj SLC13A-1
Dm Indy
Sk SLC13A5
Sp SLC13A5
Ci sialin
Tc sialin
Oj SLC17A-1
Sk sialinL
Hs SLC17A8
Hs SLC17A7
Hs SLC17A6
Dm GH23975
Hs SLC17A9
Hs SLC17A3
Hs SLC17A1
Hs SLC17A4
Hs SLC17A2
Hs SLC17A5
100
95
99
71
98
98
71
100
54
52
50
100
52
99
51
50
99
50

h

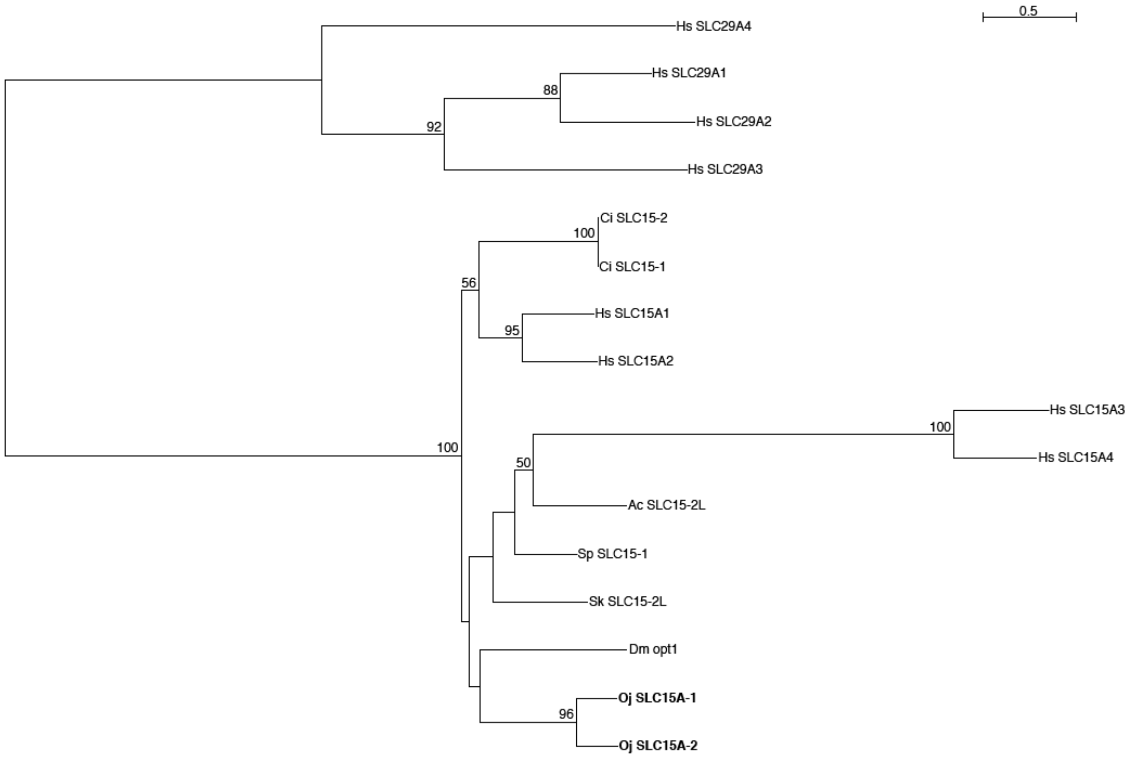
0.5
Hs SLC29A4
Hs SLC29A1
88
Hs SLC29A2
92
Hs SLC29A3
Ci SLC15-2
100
Ci SLC15-1
56
Hs SLC15A1
95
Hs SLC15A2
Hs SLC15A3
100
100
Hs SLC15A4
50
Ac SLC15-2L
Sp SLC15-1
Sk SLC15-2L
Dm opt1
Oj SLC15A-1
96
Oj SLC15A-2

i

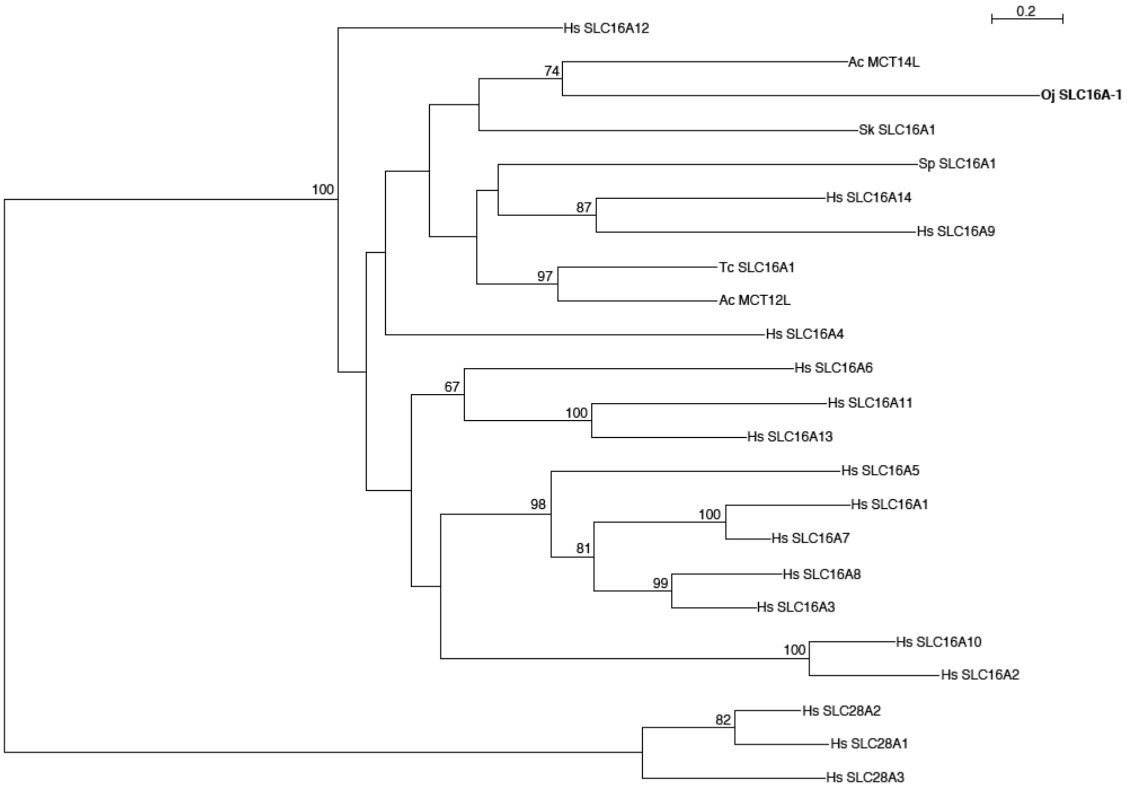
0.2
Hs SLC16A12
Ac MCT14L
74
Oj SLC16A-1
Sk SLC16A1
Sp SLC16A1
100
Hs SLC16A14
87
Hs SLC16A9
Tc SLC16A1
97
Ac MCT12L
Hs SLC16A4
Hs SLC16A6
67
Hs SLC16A11
100
Hs SLC16A13
Hs SLC16A5
98
Hs SLC16A1
100
Hs SLC16A7
81
Hs SLC16A8
99
Hs SLC16A3
Hs SLC16A10
100
Hs SLC16A2
Hs SLC28A2
82
Hs SLC28A1
Hs SLC28A3

Figure S3. Analysis of gene ontology.

The numbers at the nodes are bootstrap values (only those ≥ 50% are shown). Ac, *Aplysia californica*; Bb, *Branchiostoma belcheri*; Bf, *Branchiostoma floridae*; Cg, *Crassostrea gigas*; Ci, *Ciona intestinalis*; Ct, *Capitella teleta*; Dm, *Drosophila melanogaster*; Hs, *Homo sapiens*; Lg, *Lottia gigantea*; Mm, *Mus musculus*; Nv, *Nematostella vectensis*; Oj, *Osedax japonicus*; Pf, *Pinctada fucata*; Sk, *Saccoglossus kowalevskii*; Sp, *Strongylocentrotus purpurtus*; Tc, *Tribolium castaneum*.
